# Multi-Site Simultaneous Time-Resolved Photometry with a Low Cost Electro-Optics System †

**DOI:** 10.3390/s17061239

**Published:** 2017-05-30

**Authors:** Forrest Gasdia, Aroh Barjatya, Sergei Bilardi

**Affiliations:** 1 Space and Atmospheric Instrumentation Lab, Center for Space and Atmospheric Research, Embry-Riddle Aeronautical University, Daytona Beach, FL 32114, USA; aroh.barjatya@erau.edu (A.B.); bilardis@my.erau.edu (S.B.); 2 Ann & H. J. Smead Department of Aerospace Engineering Sciences, University of Colorado Boulder, Boulder, CO 80309, USA

**Keywords:** small telescopes, photometry, small satellites, unresolved object characterization, multi-site observation, space situational awareness

## Abstract

Sunlight reflected off of resident space objects can be used as an optical signal for astrometric orbit determination and for deducing geometric information about the object. With the increasing population of small satellites and debris in low Earth orbit, photometry is a powerful tool in operational support of space missions, whether for anomaly resolution or object identification. To accurately determine size, shape, spin rate, status of deployables, or attitude information of an unresolved resident space object, multi-hertz sample rate photometry is required to capture the relatively rapid changes in brightness that these objects can exhibit. OSCOM, which stands for Optical tracking and Spectral characterization of CubeSats for Operational Missions, is a low cost and portable telescope system capable of time-resolved small satellite photometry, and is field deployable on short notice for simultaneous observation from multiple sites. We present the electro-optical design principles behind OSCOM and light curves of the 1.5 U DICE-2 CubeSat and simultaneous observations of the main body of the ASTRO-H satellite after its fragmentation event.

## 1. Introduction

Many resident space objects (RSO) in Earth proximity consist of materials which reflect sunlight. This reflected sunlight signal is often visible to observers on the ground and has been used for tracking and subsequent orbit determination of satellites and space debris since the beginning of the space age [1,2]. The United States Space Surveillance Network uses these optical observations to supplement radar data for locating and tracking objects in space [3]. Optical observations are usually limited to just a few hours a night before dawn and after dusk. As the RSO rotates, and/or the observer-RSO-sun angle changes, the reflected sunlight signal changes. Thus, in addition to providing astrometric orbit determination, this passive signal also contains high fidelity geometric information about the object [4]. For RSOs that cannot be imaged directly because they are too small or distant to be resolved, analysis of photometric light curves provides insight into their physical state; this includes small satellites, CubeSats, or debris in low Earth orbit, or large satellites out at geosynchronous orbit. Analysis of this photometric data can provide information to observers about size, shape, material makeup, and attitude [5].

Astronomers and planetary scientists have developed techniques for estimating the size, shape, and albedo of asteroids from photometric light curves [6,7,8]. These techniques typically require two to three months of observations to view the asteroid from a sufficient range of angles and lighting conditions in order to fit a model of the asteroid’s shape and spin axes. Similar approaches can be applied to satellites and space debris, although lighting and viewing geometry change faster because they are closer to Earth. Additionally, while asteroids are roughly round, smooth, and have stable rotations, the opposite is true of man-made space objects, which often have sharp edges and flat surfaces that produce distinct specular glints. Operational satellites that are 3-axis stabilized pose an additional challenge because changes in the object’s attitude are very challenging to predict using a simple physics model. To accurately characterize an RSO, especially in low Earth orbit (LEO), it is necessary to capture photometry at a much higher cadence than near-Earth asteroids or even satellites in geosynchronous orbit (GEO).

CubeSats are an interesting part of the space debris problem because they can be considered both functional satellites and unmaneuverable debris. Because of the low cost of entry, rapid development cycle, and increasing capability of microelectronics, there has been exponential growth in the number of CubeSats launched yearly. CubeSats are now being launched by educational institutions ranging from universities to elementary schools, as well as a number of developed and developing countries [9,10]. Unfortunately, more than one in three CubeSats that reach orbit suffer mission failure [11]. Often, little is known about specific causes of failure because the most common reason for declaring a failed mission is no-contact with the satellite followed by power or communication loss. In such cases, ground-based photometry can not only be used to measure CubeSat spin rate, and hence controlled or uncontrolled nature of the CubeSat, but also determine if an antenna or solar panel has deployed, or identify individual CubeSats deployed in a group [12]. When time-resolved photometry is used in an operational mission support role, prior knowledge on the satellite’s shape, materials, and deployables provide increased confidence when estimating the satellite’s physical state compared to when both the shape and attitude are unknown [13].

Embry-Riddle’s Daytona Beach campus has been developing a low cost and portable system called OSCOM that consists primarily of commercial-off-the-shelf (COTS) hardware capable of high-cadence photometric observations of CubeSat-sized RSOs [14]. OSCOM stands for Optical tracking and Spectral characterization of CubeSats for Operational Missions. As the name implies, OSCOM’s primary purpose is to produce time-resolved photometry of CubeSats and other small satellites to provide mission support to satellite operators, but it can also support other space situational awareness (SSA) objectives. Just as cheap micro-electronics and standardized CubeSat form factor has made access to space easy for all educational institutions and other countries, our objective for OSCOM was to enable CubeSat enthusiasts to do high cadence multi-point observations of their CubeSats as part of mission support activities at a relatively low cost—less than USD 10K total for optics, camera, mount, and accessories.

**Figure 1:**
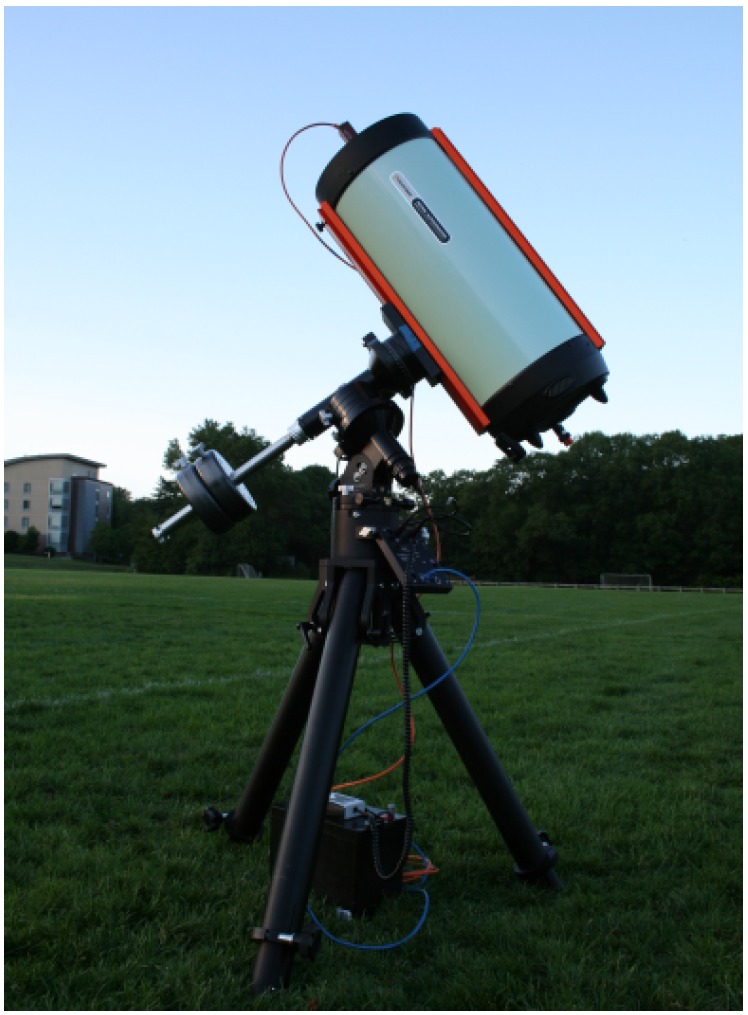
OSCOM telescope system for small satellite and debris photometry deployed in Needham, MA, USA. Used with permission from Gasdia et al. [14] ^©^ 2016 AMOS Conference.

OSCOM utilizes a COTS 11-inch aperture telescope and CMOS machine vision camera (see Figure 1) for a low cost and portable design. The system can be easily deployed for simultaneous multi-point observations of satellites. In particular, by deploying OSCOM telescopes at separate sites up to a hundred miles apart, space objects can be observed under two different observation geometries simultaneously. This multi-point observation mode provides additional information to do rapid state estimation of space objects.

This paper first presents electro-optical system design and radiometry for RSO observation, then introduces OSCOM specifics, and finally presents sample photometry demonstrating OSCOM’s performance, including multi-point simultaneous observation of the main body of the Japan Aerospace Exploration Agency (JAXA) ASTRO-H satellite after it broke apart in orbit.

## 2. Satellite Brightness Theory

The visual magnitude $m_{v}$ of an RSO illuminated by the sun is found by taking the incident solar flux and reflecting it to an observer. In magnitude space, this process can be approximately described by the following Equation:

(1) $$m_{v}=\underset{1}{\underset{︸}{-26.75}}+\underset{2}{\underset{︸}{5\log(R)}}-\underset{3}{\underset{︸}{2.5\log(\rho A)}}-\underset{4}{\underset{︸}{2.5\log\left(F(\Phi )\right)}},$$

where -26.75 is the visual magnitude of the sun from the earth [15], *R* is the observer-to-satellite distance, ρ is the albedo, *A* is the cross-sectional area of the RSO, and F(Φ) is a phase function that depends on the relative orientations of the observer, reflecting surface, and sun [16]. Atmospheric extinction and other effects reduce the actual brightness that is observed from the ground. The first term, the solar magnitude, represents the incident flux on the satellite in the photometric *V* band centered on 550 nm. Term 2 is just the inverse square law of irradiance and shows that every doubling in satellite-observer distance increases the apparent magnitude (decreases the brightness) of the RSO by 1.5 magnitudes. Terms 3 and 4 carry the optical signature information that is unique to each satellite. Term 3 represents the albedo-area product [17]. Each reflecting facet on the satellite has an area and characteristic albedo that cannot be separated from brightness observations alone. For a fixed albedo, every doubling in surface area increases the brightness by 0.75 magnitudes. The fourth term is the most complex and contains shape and attitude information as a function of the phase angle. Satellites are typically made up of multiple materials and have distinct shapes that each have their own values for the last two terms. For unresolved observations, each reflecting facet contributes to the overall observed brightness and are summed in the equation. Even physically small changes in satellite geometry can have a meaningful impact on the observed magnitude.

For RSO observations, the phase angle is defined as the observer-RSO-sun angle and has a value of 0° when at full phase. When described with respect to the RSO body, the phase angle can be broken up into four angles that uniquely describe the illumination geometry: a latitude and longitude to describe the position of the sun and a latitude and longitude to describe the position of the observer. This four-angle description is necessary for recovering detailed physical information about an RSO from its light curve [18]. Assuming the angles are known, McCue et al. [19] present a table of phase functions for diffuse and specular reflections from several simple shapes. Specular flashes from flat plates can be orders of magnitude brighter than any diffuse reflector of the same area and are potentially useful markers in a satellite’s optical signature. Additionally, because of the small angular extent of the sun when viewed from about 1 AU, the specular reflection is sensitive to the angle of surface-normal to less than 1°. Therefore, specular glints can be very brief, especially for rotating objects in low Earth orbit. In order to accurately capture them in time-resolved photometry, high cadence measurements are crucial. For spin-stabilized satellites with some symmetry in their physical structure, the specular reflections off the satellite surface will be an integer multiple of the spin rate. Thus, to sufficiently resolve a complex satellite with a high degree of symmetry, at least several Hz photometry is required.

Simultaneous observation of an RSO from two different sites can be used to constrain estimates of the object’s attitude and provide range information through parallax. Multi-site simultaneous observations are particularly useful in the interpretation of 3-axis controlled or slowly rotating objects in LEO, which often present only a single face during a pass. Hall et al. [13] present a simple simulation that demonstrates how simultaneous observations from Maui and Kauai, Hawaii can distinguish between LEO single-facet signatures caused by changes in illumination versus changes in visibility.

## 3. System Design Fundamentals

Optical characterization of small satellites and debris in LEO is challenging because small objects have low signal levels that typically require long exposure times for sufficient signal-to-noise ratio (SNR), but, in order to temporally resolve rapid changes in RSO brightness, exposure times are limited. As the primary goal of OSCOM is characterization rather than orbit determination or cataloging of new space debris objects, the OSCOM telescope system points at and tracks the RSO as it passes overhead. Although background stars will appear as streaks in the images, the RSO remains fixed in the field of view during the integration time, appearing as a point source and maximizing the SNR. This section explains the electro-optical system design fundamentals behind OSCOM. Shell [20], Ackermann et al. [21], and others provide a more detailed development of the theory of optical RSO observation.

### 3.1. Radiometry

Photons from both the RSO and the background sky are collected by the telescope and converted to electrons by the image sensor. Although the flux from the RSO is eventually isolated from the background during the photometry process, the background sky contributes noise to that measurement and is therefore considered during the design process. Small satellites in LEO or large satellites at GEO are optically unresolved for telescopes smaller than 1 m aperture. A 10 cm object at 1000 km range subtends only 0.1 μrad while the Rayleigh criterion minimum resolvable angle for a 1 m telescope in green light is 0.65 μrad, so we model the object as a point source. The background sky fills the telescope field of view so it is modeled as an extended source.

One can estimate the brightness of a 1 U (10 cm ×10 cm ×10 cm) CubeSat using Equation (1) and the phase functions from McCue et al. [19]. At a slant range of 1000 km, the diffuse reflection from a full face of the CubeSat with albedo 0.2 and at full phase is only magnitude 11.2 before atmospheric extinction. The photon irradiance of an RSO given its magnitude *m* can be estimated with

(2) $$E_{\mathrm{RSO}}\approx 6\times {\mathrm{10}}^{10}\times 10^{-0.4m}\;\left[\mathrm{ph}/s/m^{2}\right],$$

where the conversion factor of 6×10^10^ is the photon irradiance of the zero-magnitude star Vega integrated over the typical spectral range of a silicon-based sensor. The 1 U CubeSat with an exoatmospheric magnitude of 11.2 corresponds to about 2×10^6^ $\mathrm{ph}/s/m^{2}$, but the atmosphere attenuates the RSO signal by much as 1 magnitude depending on the airmass the RSO is being observed through.

**Figure 2:**
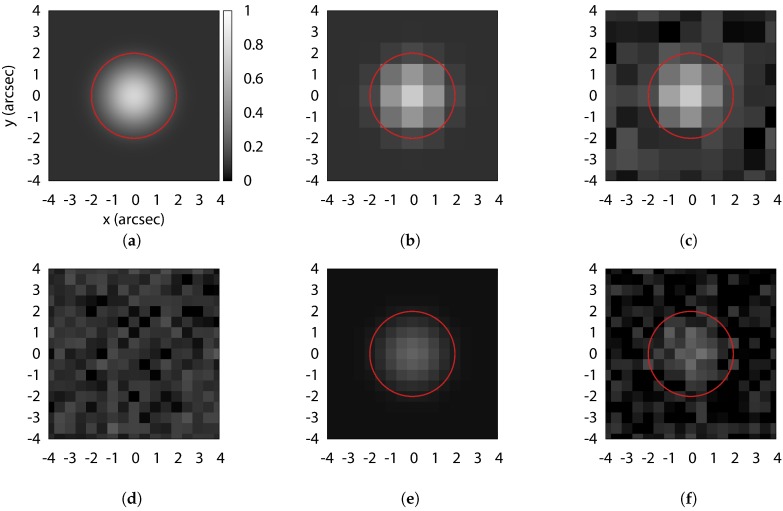
Effect of sampling a two-dimensional Gaussian of 2 arcsec FWHM (**a**) by image scales 1 arcsec/pix (**b**) and 0.5 arcsec/pix (**e**) and with source Poisson and Gaussian read noise (**c**,**f**). Sample detector read noise is shown in (**d**). Red circles represent 2 arcsec radii photometric apertures. All figures are at the same spatial and intensity scale.

The photon flux of a point source is a function only of the aperture area *A* and distance from the optical system to the source. If all of the RSO signals were collected by a single detector pixel, the number of photoelectrons read out would be

(3) $$e_{\mathrm{RSO}}=\mathrm{QE}\tau AE_{\mathrm{RSO}}t\;\left[e^{-}\right],$$

for detector quantum efficiency QE, optical transmission efficiency τ, and integration time *t*, but the RSO signal is typically spatially sampled by the detector pixels. When viewed through a turbulent atmosphere, the RSO is imaged onto the focal plane as a spot which is the convolution of the atmospheric seeing and the point spread function (PSF) of the optical system. The resulting intensity distribution of a point source through atmospheric turbulence is approximately Gaussian with a full width half maximum (FWHM) related to wavelength and Fried’s parameter, $r_{0}$, a measure of atmospheric turbulent stability [22]. Typical seeing disks vary from 1 to 3 arcsec FWHM depending on site conditions and is independent of the telescope aperture. However, the seeing disk increases at lower elevation angles. Although astronomical observations typically occur at low airmass, optical satellite observations often follow the satellite to lower elevation angles to capture data over a wider range of phase angle. It is important to consider this larger seeing disk when designing the system. Because seeing smears the point source into a relatively large area, only a portion of the total light collected by the optical system is sampled by individual pixels of the detector (see Figure 2). With 2.5 arcsec seeing and a perfect 1 m telescope at focal ratio f/D=8.6, the FWHM RSO spot size on the detector is 104 μm, much larger than the diffraction limited spot and much larger than typical charge-coupled device (CCD) or complementary metal–oxide–semiconductor (CMOS) image sensor pixels.

Radiometry of the background sky depends not only upon the aperture, but the focal length of the optical system. As the field of view increases, background sky photons are collected from a larger area of sky. The number of background sky photoelectrons read out from each pixel is

(4) $$e_{b}=\mathrm{QE}\left(\frac{\tau \pi L_{b}A_{\mathrm{pix}}}{1+4{\left(f/D\right)}^{2}}\right)\times t\;\left[e^{-}\right],$$

where pixels have collecting area $A_{\mathrm{pix}}$, *f* is the focal length, and *D* is the diameter of the aperture [23]. $L_{b}$ is the radiance of the background sky. For an urban sky background of 18.5 $\mathrm{mag}/{\mathrm{arcsec}}^{2}$, the photon radiance is about 10^14^ $\mathrm{ph}/s/m^{2}/\mathrm{sr}$.

### 3.2. Photometric Signal-to-Noise Ratio

In circular aperture photometry for an array detector, the brightness of a source is measured by adding up all of the detector digital counts from pixels which fall inside of a circular area on the detector. The number of digital counts in each pixel of the image is related to the number of source photons detected by the pixel, the number of background photons detected by the pixel, and noise that occurs during the readout process of the detector. See Howell [24], Howell [25], or Mighell [26] for details on the photometry process.

Although each pixel can be considered an individual measurement, the overall SNR of the aperture photometry considers the RSO signal summed over a group of pixels. The signal to noise ratio for circular aperture photometry is described by

(5) $$\mathrm{SNR}=\frac{e_{\mathrm{RSO}}^{*}}{\sqrt{e_{\mathrm{RSO}}^{*}+n_{\mathrm{pix}}\left(e_{b}+{\mathrm{RN}}^{2}\right)}},$$

where  $e_{\mathrm{RSO}}^{*}$ is the portion of $e_{\mathrm{RSO}}$ captured by the photometric aperture, i.e., encircled energy, $n_{\mathrm{pix}}$ is the number of pixels in the aperture, and RN is the read noise of the detector. As the photometric aperture size increases, more RSO signal is counted, but the contribution of sky background and read noise also increases. The SNR is maximum at fairly small photometric aperture radii, approximately equal to 0.68 FWHM of the RSO intensity profile [24]. However, because of the likelihood of centering errors, and the relatively slow decrease in SNR around the 0.68 FWHM radius, aperture radii of 1 FWHM are typically a better choice.

To minimize the read noise contribution, we want the RSO spot to be sampled by as few pixels as possible. Although the maximum energy density would be obtained by putting nearly all of the RSO light in one pixel, this results in loss of information about image structure and the centroid of the RSO. Harris [27] shows that critical sampling is achieved when the FWHM is approximately two pixels. The pixel scale is simply x/f where *x* is the linear dimension of the the pixel and *f* is the effective focal length of the optical system.

For an RSO much brighter than the sky background and read noise, the SNR scales with $\sqrt{t}$. Newberry [28] shows that if the sky background is the dominant noise source, then the SNR also increases as $\sqrt{t}$. If the detector noise is the dominant noise source, then the SNR will be lower than the sky-limited case, but grows faster than $\sqrt{t}$. Therefore, the exposure time should be at least long enough such that sky noise is greater than the read noise of the detector. This puts a limit on the minimum exposure time that should be used for time-resolved photometry of dim targets.

### 3.3. Design Trades

**Figure 3:**
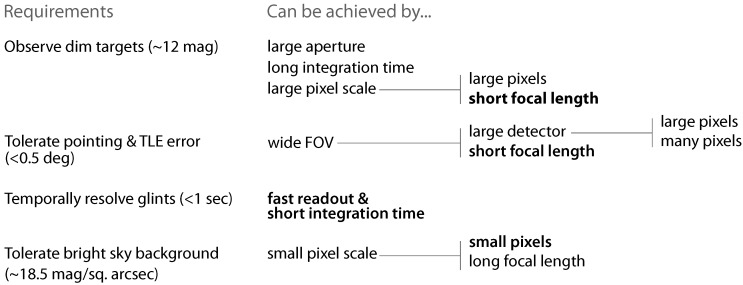
Electro-optical system requirements and design options for observing small satellites and debris. Text in bold represents options chosen by the OSCOM system.

The telescope aperture, focal length, and detector pixel and array size each need to be determined for developing a system that is capable of observing small satellites and debris using low cost COTS equipment. General requirements and design options are summarized in Figure 3. There are several ways to achieve similar results and additional practical factors, e.g., optics transmission efficiency, focus and collimation, and detector noise, also effect the performance of any real system.

**Figure 4:**
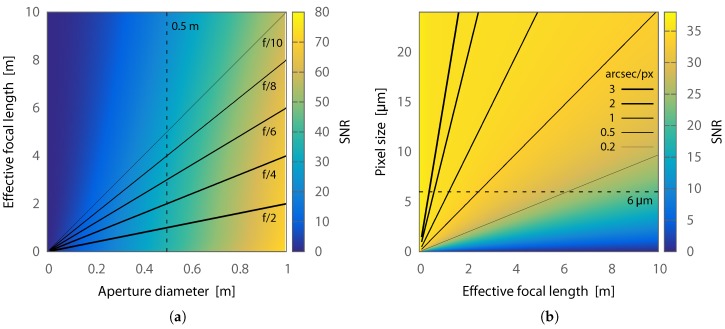
(**a**) aperture diameter, a proxy for area, has the strongest influence on signal-to-noise ratio (SNR) of an unresolved RSO. However, for a given aperture, there is also an improvement in SNR at shorter focal lengths. This plot assumes 6 μm pixels; (**b**) oversampling an unresolved RSO spot reduces the photometric SNR by increasing the sky background and read noise for a constant 1 FWHM photometric aperture radius. This plot assumes a 0.5 m aperture.

Figure 4 shows the simulated SNR of an unresolved RSO by varying the optical aperture diameter, focal length, and pixel size of two similar sample systems. The SNR is calculated for a 14th magnitude RSO with a 19 $\mathrm{mag}/{\mathrm{arcsec}}^{2}$ sky background, an optics transmission efficiency of 0.8, and a detector with an average QE of 0.5, read noise of $6e^{-}$, and integration time of 0.125 s. We assume an RSO spot size of 3 arcsec, including seeing and tracking jitter, and use a near-optimal 1 FWHM photometric aperture radius. Figure 4a assumes a detector with 6 μm pixels and demonstrates that increasing aperture is the fastest way to increase SNR. However, for a given aperture diameter, the SNR is higher at shorter focal lengths because fewer pixels are being included in the photometric aperture, and therefore the total read noise and background sky contribution is reduced. Figure 4b assumes a 0.5 m aperture diameter and shows that larger pixel scales result in increased SNR. In practice, the pixel scale should be at least 2 pix/FWHM to avoid undersampling issues.

Although it does not directly impact the SNR, in practice, we have found that a large field of view is of immense benefit when tracking small satellites and debris. Infrequently updated or relatively low accuracy orbital elements and telescope pointing error creates uncertainty in the predicted time and sky location of acquisition by the tracking system. If acquiring an LEO RSO 20° above the horizon, a 1° field of view allows for up to about 2 s in along-track error. Field of view can be increased by using a shorter focal length or a physically larger sensor array, but there are practical limitations to both of these parameters. It is difficult to manufacture quality large aperture, short focal length optical systems at low cost, and for a given pixel size that matches the optical system and site seeing conditions, a  larger sensor array requires more pixels. Transferring data from more pixels slows the frame rate, but in practice, the rate is still fast enough for dim RSOs and a portion of the detector pixels can usually be read out from a region of interest at increased speeds. Unfortunately, image sensors with an increased number of pixels will likely be more expensive than an otherwise identical sensor with fewer pixels.

The ideal electro-optical system for time-resolved RSO photometry would have a large aperture, low focal ratio and many pixels for a large field of view, and the proper pixel size for critical sampling of the seeing disk, but available COTS equipment puts the ultimate limit on what can be achieved at low cost.

## 4. OSCOM System Components

In its most basic form, the OSCOM telescope system for small satellite photometry consists of a tracking mount, telescope optics, and CMOS image sensor. The tracking mount moves the telescope along the two-line element set (TLE)-predicted path of an RSO through the sky and the CMOS imager records images of the unresolved satellite and sends them to a computer where they are saved to a solid state drive. Besides meeting the general requirement of time-resolved photometry of small satellites and debris, the OSCOM electro-optical system also had to be portable and low cost in order to support simultaneous observations from multiple sites or in multiple optical bands. This was accomplished using COTS hardware; the system components are listed in Table 1.

OSCOM was developed for observations from Daytona Beach, FL, USA, and, therefore, the system components were chosen to match conditions in an urban sea-level environment. The bright sky background and poor seeing conditions of Daytona Beach are a near worst-case scenario for dim source photometry. Nonetheless, the OSCOM system is capable of 1 U CubeSat photometry at several Hz from Daytona Beach.

### 4.1. Image Sensor

To obtain high temporal resolution photometry, OSCOM has chosen to use CMOS image sensors with global shutters. Because of their active pixel sensor (APS) design, CMOS sensors read out many times faster than CCDs with an equivalent number of pixels. Additionally, a tremendous amount of research has been devoted to manufacturing CMOS image sensors in cell phones and similar devices. Because of this, CMOS imagers marketed for machine vision applications with less than 6 e^-^ of read noise are available for under $1500. These types of cameras typically only have 12 bit analog-to-digital converters, which limits their dynamic range, but scientific CMOS (sCMOS) cameras are available with 16 bit ADCs and read noise as low as 1 e^-^ for about $10,000 [29].

The Manta G-235 imager (Allied Vision, Exton, PA, USA) used by OSCOM since 2015 was one of the first COTS sensors with the Sony IMX174 CMOS. The IMX174 was Sony’s first sensor to use Pregius technology, which provides higher dynamic range, quantum efficiency, and lower read noise in global shutter CMOS sensors than similar CCDs [30]. The IMX174 is now used in cameras made by several companies, and Sony produces multiple image sensors with Pregius. With rapid developments in image sensor technology and their relatively low cost, we plan to reevaluate which sensor to use with OSCOM every few years.

### 4.2. Telescope

As discussed in Section 3.2, the optimum SNR is obtained by matching the pixel scale to the seeing conditions of the observatory site. In Daytona, the OSCOM site often has 2.5 to 3 arcsec seeing. Critical sampling then calls for approximately 1.5 arcsec/pix. In practice, there is additional motion blur from the tracking mount, so we were interested in pixel scales between 1.5 and 2 arcsec/pix.

Classical telescope designs typically have focal ratios of f/8 or higher. For even a half meter aperture telescope of this focal ratio, 39 μm pixels are required for 2 arcsec/pix sampling. Figure 4b shows the pixel scales that can be obtained for different combinations of effective focal length and pixel size. COTS detectors, especially CMOS, are typically limited to 12 μm pixels or smaller, although binning can increase the effective size if the camera is capable. To accomplish 2 arcsec/pix sampling with a 0.5 m telescope and 6 μm pixels, the telescope optic must have an effective focal ratio of only f/1.24. No COTS optical tube is available that meets these specifications.

One of the only low cost, small to mid-aperture, low focal ratio COTS optical tubes is the Rowe Ackermann Schmidt Astrograph (RASA) made by Celestron (Torrance, CA, USA). The RASA is available in 11 inch aperture, but limited numbers of a 14 inch version are available from Celestron for SSA applications. The design achieves a wide, well corrected image field at f/2.22 by replacing the secondary mirror of a typical Schmidt Cassegrain with a corrector lens assembly. The detector is mounted at the focus of the primary mirror. Ackermann et al. [31] presents an analysis of the sensitivity and search rate of four dozen COTS optical tubes for SSA applications. Although they focused on systems for detecting small RSO, and OSCOM focuses on characterization of specific RSOs, Ackermann et al. [31] comes to the same conclusion—that the RASA telescopes are excellent value for their performance.

Specifications for the OSCOM electro-optical system pairing an 11 inch RASA with the Manta G-235 image sensor are shown in Table 2. The pixel scale satisfies our requirement of 1.5 to 2 arcsec/pix and the field of view is sufficiently large for tracking LEO satellites with known orbital elements. Although the RASA can be used with a larger image sensor format, the improved sensitivity of the IMX174 was prioritized over field of view during the OSCOM design process.

### 4.3. Tracking Mount

The tracking mount is the third major OSCOM component. There are few telescope mounts inside the $10,000 budget that advertise the capability to do satellite tracking, but we have found that many mounts can be adapted for this use. Amateur telescope mounts are typically either of the altitude-azimuth or German equatorial style. Altitude-azimuth mounts are able to continuously track most LEO satellites through the sky, except for passes that are almost directly overhead. The design of most amateur altitude-azimuth mounts would require them to near-instantaneously rotate 180° in azimuth for a satellite passing through zenith. An equatorial wedge can be utilized to move the pole from zenith to some other elevation angle. This is advantageous because airmass is at a minimum at zenith and data there is often of the best quality during a pass. German equatorial mounts are aligned to the celestial pole so that only one motor is required for tracking a star field. When tracking satellites, motors in both axes are used. Although German equatorial mounts do not suffer from the pole at zenith when the satellite track is mostly parallel to the meridian, this mount type is unable to continuously track an object that crosses from one side of the meridian to the other. Unfortunately, many of the higher quality, advanced amateur telescope mounts are of the German equatorial design.

The minimum requirements for an LEO satellite tracking mount is the ability to carry the telescope and imager payload at a rate of more than 2 to 3°/s. These rates provide ample margin for tracking an object in an International Space Station-like orbit through zenith. Less expensive mounts usually exhibit greater tracking jitter when moving at these rates, which should be taken into consideration when determining what pixel scale should be used. OSCOM has demonstrated several amateur telescope mounts for LEO satellite tracking, including the Losmandy G-11 (Burbank, CA, USA). This mount is sized for the 11 inch RASA and is easily modified by the user to extend the ability to track through the meridian or upgrade the worm gear and housing. OSCOM has also used Software Bisque Paramount MX and ME mounts (Golden, CO, USA), which are several times more expensive than the G-11 but provide smoother tracking at high rates.

**Figure 5:**
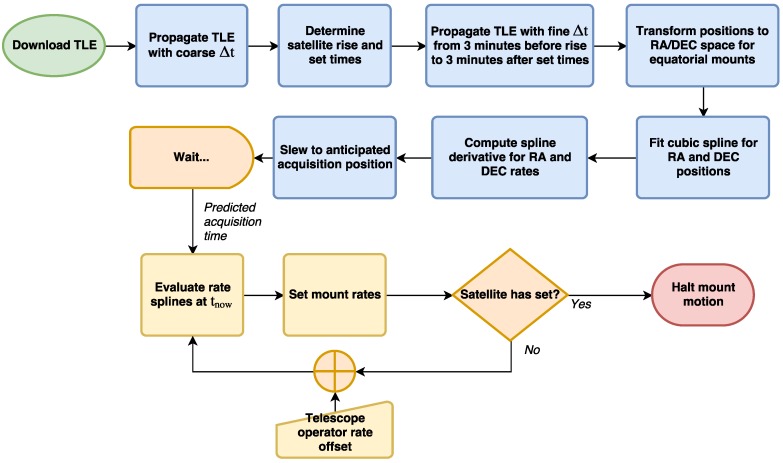
An open/human-in-the-loop algorithm for satellite tracking by two-line element set (TLE).

Each mount used by OSCOM was easily adapted for satellite tracking because software control interfaces are available to the user. The Paramount control software, TheSkyX, natively supports satellite tracking and an improved satellite tracking add-on is available for purchase from Software Bisque, but custom tracking programs provide more flexibility. All that is required is writing a program that updates the slew rates of the mount to match the angular rates of the satellite as it passes overhead. A simple open loop algorithm used by OSCOM is shown in Figure 5. Open loop control assumes that the mount alignment, computer clock time, and TLE are perfectly accurate. The telescope operator can provide manual offsets to the TLE-derived tracking rates if necessary. Although an open loop controller is sufficient for carefully aligned telescope mounts and satellites with regularly updated TLEs, a closed loop controller with machine vision feedback is preferred for fully automated observations and satellites with old or low confidence orbital elements. In the future, we intend to present a version of OSCOM that automatically corrects for mount alignment and TLE errors with machine vision optical feedback.

## 5. Operation

In its current form, OSCOM is deployed by a single telescope operator. The whole setup process takes less than an hour for an experienced operator and typically occurs just before sunset. The mount and telescope can be set up on a permanent pier for regular observations or on a tripod for portable deployments. Computer time is synced via a network time protocol (NTP) time server and the mount pointing is calibrated to alignment stars using the mount’s native multi-star alignment software. The telescope is slewed to an evenly illuminated patch of sky so flat fields can be taken. Flats allow for post processing correction of vignetting, shadows from any specks of dust on the optics, and pixel-to-pixel sensitivity variation. The mount drive is set to several times the sidereal rate so that stars appear as long streaks on the camera display, if they are visible at all. Over a hundred individual flat fields are captured using the Genika Astro (version 2, AiryLab, Gréoux les Bains, France) [32] image capture software in under a minute.

The operator brings up the TLE for the first satellite to be observed and loads it into our satellite tracking program. As described by Figure 5, a few minutes before we expect to acquire the satellite, the telescope slews to the acquisition location, typically 10 to 15° above the horizon. Tracking begins automatically at the expected time, or at the command of the operator if he or she expects along-track error in the TLE. Images are recorded and captured to a solid state drive using Genika Astro. If the satellite is drifting in the image, the telescope operator can manually adjust the mount track rates. When the satellite has entered a shadow or otherwise is no longer visible, the track is terminated and the mount stops. The operator can then load the TLE of the next satellite to be observed and the process repeats. At the end of the evening, the detector is removed from the telescope and covered with a light-tight cap so bias frames can be captured.

After observations, the images are converted from .ser to .fits format in Genika Astro and loaded into a custom multi-threaded Python library called the Optical Satellite Analysis Toolkit (OSAT). OSAT makes use of several open source libraries [33,34,35,36] to produce master bias and flat frames and reduce the images, detect the satellite in each frame, perform photometry, and then correct the light curve for satellite range, as in Equation (1), and first order atmospheric extinction. The extinction coefficient is estimated from known star fields imaged at different elevations during the observing session, but precision absolute photometry calibrated light curves are time-consuming to produce and not the typical OSCOM data product. We are beginning work on a new version of OSAT to provide calibrated magnitudes without the need to explicitly image and analyze standard star fields, but current light curve corrections are sufficient for analysis of most RSOs. Lastly, OSAT has a simple analysis module for filtering and performing periodograms on the light curve data. Additional information on the OSCOM image reduction and photometry process can be found in Gasdia [37].

## 6. Results

The OSCOM system has observed dozens of small satellites from several sites along the East coast of the United States. Two sample photometric light curves are presented below: one demonstrates temporally resolved photometry of a 1.5 U CubeSat with ten deployables and the other a simultaneous multi-site observation of the parent body of the fragmented ASTRO-H satellite. For both datasets, the raw images were reduced by first subtracting a master bias image and then dividing a normalized master flat image to correct for optical vignetting and variations in pixel-to-pixel sensitivity. Aperture photometry was performed using OSAT. The system zero point determined by known comparison stars shifts them to the magnitudes shown. Error bars represent photometry magnitude error.

One of the fundamental techniques for interpreting light curves, especially for spinning or rotating objects, is temporal spectral analysis [38]. This analysis helps quantify the frequency of periodic features of the light curve and establish the rotation rate of the object. Common techniques for determining the light curve frequency components include the fast Fourier transform (FFT) and the Lomb–Scargle periodogram [39], but we have found that these techniques assign the most power to the shortest light curve flash period and often miss the true object rotation period completely. Others have had similar experiences [40,41]. The Plavchan periodogram is a phase dispersion minimization (PDM) method that accepts unequally sampled data [42]. Unlike FFT-based methods, it does not assume sinusoidal variations and we have found that it usually does a better job at identifying the fundamental period at the cost of being noisier overall. As a check on the fundamental frequency, the light curve is folded onto itself and plotted as a function of the fundamental phase. If the periodogram determined an incorrect period, the phase diagram will visually appear very noisy, whereas if it determined the correct period, the light curve should be clearly visible.

### 6.1. DICE-2 (37852)

**Figure 6:**
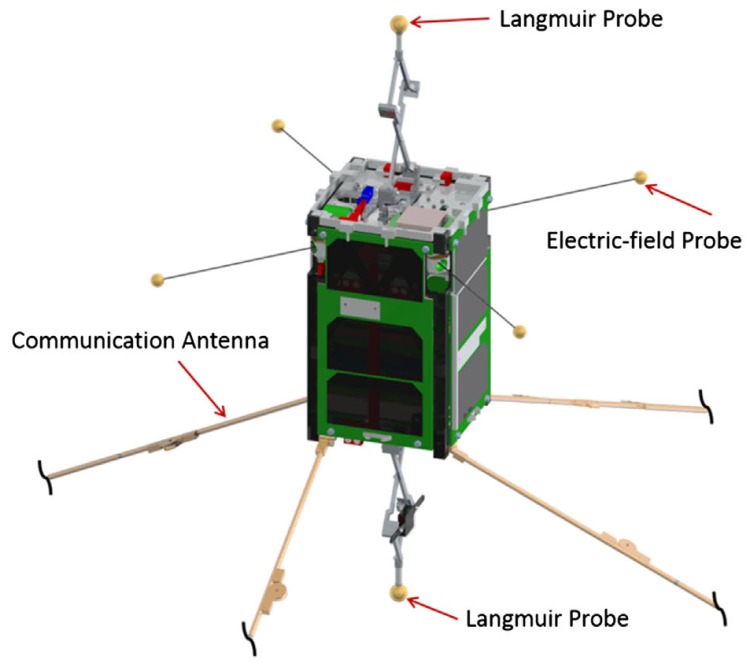
Diagram of the DICE CubeSat with deployables. Figure from Fish et al. [44].

**Figure 7:**
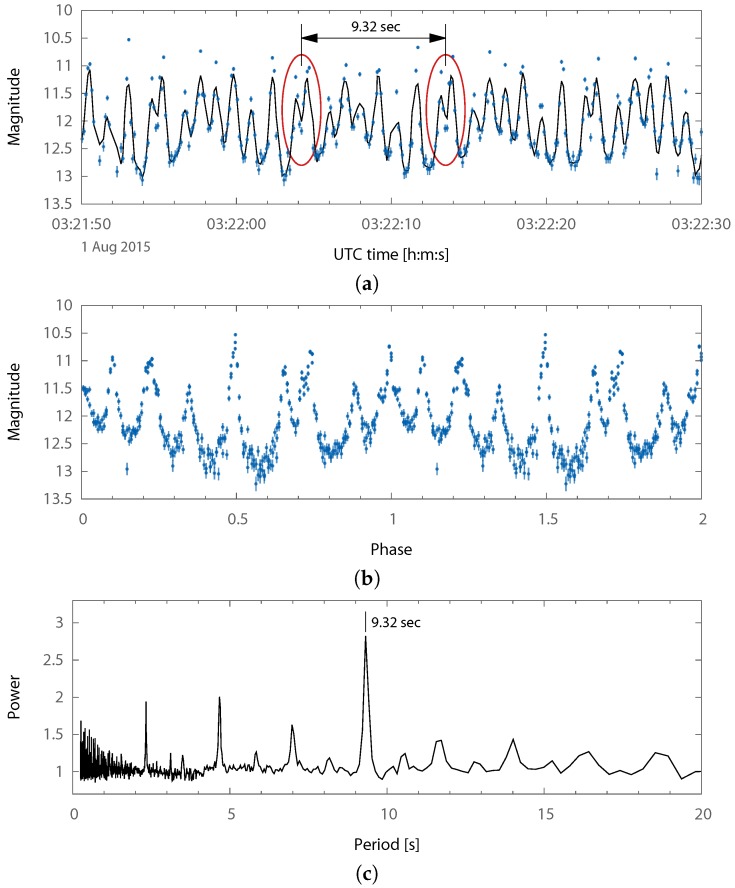
(**a**) light curve of the DICE-2 CubeSat (Spacetrack ID 37852) spinning at about 0.1 Hz. This observation occurred using the OSCOM system shown in Figure 1 from Needham, MA, USA using 125 ms integration time. The smoothing line is a five-point centered moving average. (**b**) phase diagram with five 9.32 s periods of the light curve from 03:21:45 UTC to 03:22:31 UTC overplotted. (**c**) Plavchan periodogram of the DICE-2 light curve showing a primary period of 9.32 s.

DICE-2 is one of a pair of 1.5 U CubeSats in the Dynamic Ionosphere CubeSat Experiment. The DICE mission is a collaborative effort funded by the National Science Foundation with the goal of mapping the geomagnetic storm enhanced density plasma bulge and plume formations in Earth’s ionosphere [43]. DICE-2 was inserted into a 456 km ×807km orbit, but had circularized by atmospheric drag into a 437 km × 682 km orbit at the time of observation. Although it was last known to be operational in 2014, the planned final spin stabilized rate was 0.1 Hz [44]. Despite the small size of the spacecraft bus, it has ten deployable booms that create specular glints (see Figure 6). Figure 7 shows a consistent flash pattern with a period of 9.32 s, approximately equal to the expected spin rate. Communication was dropped due to a spacecraft electronics anomaly attributed to the space environment [43]. However, it is possible that the onboard attitude determination and control electronics and algorithms continued to work and eventually achieved the ∼0.1Hz that we observe from the ground nearly one year after contact was lost.

DICE is an excellent example of the need for temporally resolved small satellite photometry. Using conventional CCD imagers which limit the imaging cadence to about 1 s, the DICE-2 flash pattern in the photometric curve would have been significantly aliased, or possibly averaged out if longer exposure times were used.

### 6.2. ASTRO-H (41337)

**Figure 8:**
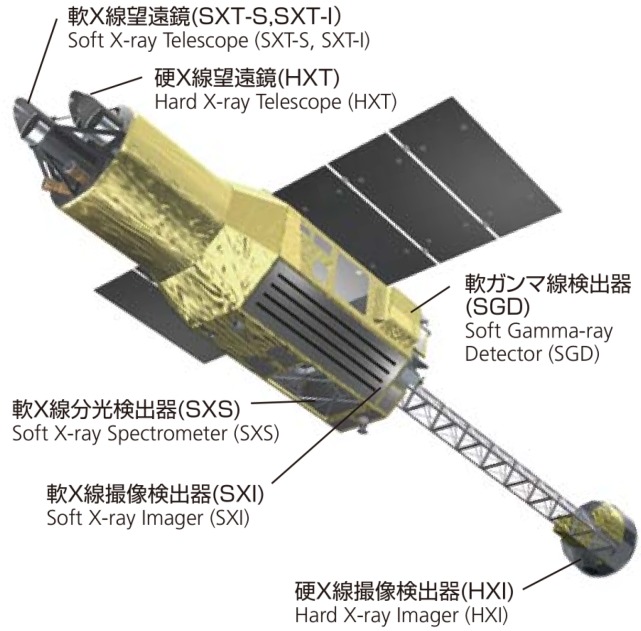
Diagram of the JAXA ASTRO-H X-ray astronomy satellite in its configuration before an on orbit fragmentation event. Figure adapted from JAXA [47], Courtesy of JAXA, ^©^ JAXA.

**Figure 9:**
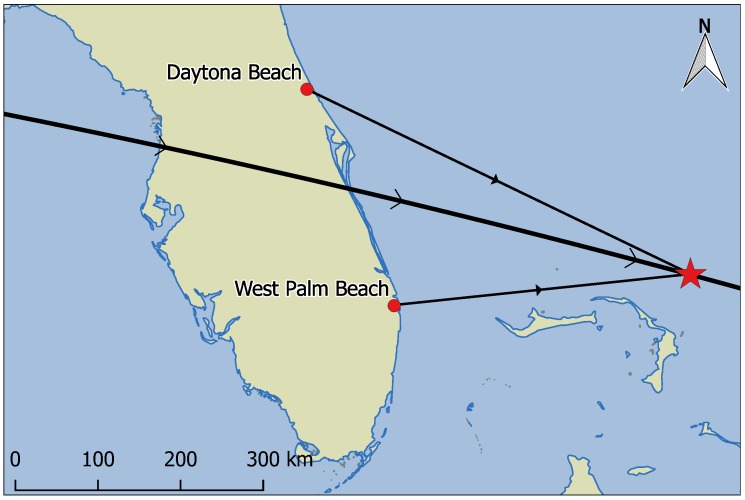
Geometry and ground track of the simultaneous observation of ASTRO-H Object A from Daytona Beach and West Palm Beach, FL on 7 May 2016. Object A at 09:31:05 UTC is marked by the star.

ASTRO-H, also known as Hitomi, was a Japanese Aerospace Exploration Agency (JAXA) X-ray astronomy satellite (shown in Figure 8) that had a catastrophic attitude control system failure in March 2016 [45]. The spacecraft spun itself apart into approximately eleven primary pieces including the parent body, many of which were tracked by the OSCOM system. Object A (Spacetrack ID 41337), the parent body and largest of the pieces, was observed simultaneously by identical OSCOM tracking systems located in Daytona Beach and West Palm Beach, FL, USA (see Figure 9). The image capture computer at each site was synced to an NTP time server before the observation. NTP typically provides timing accuracy to much better than 0.1 s [46], but the precise timing error between the two sites was never quantified.

**Figure 10:**
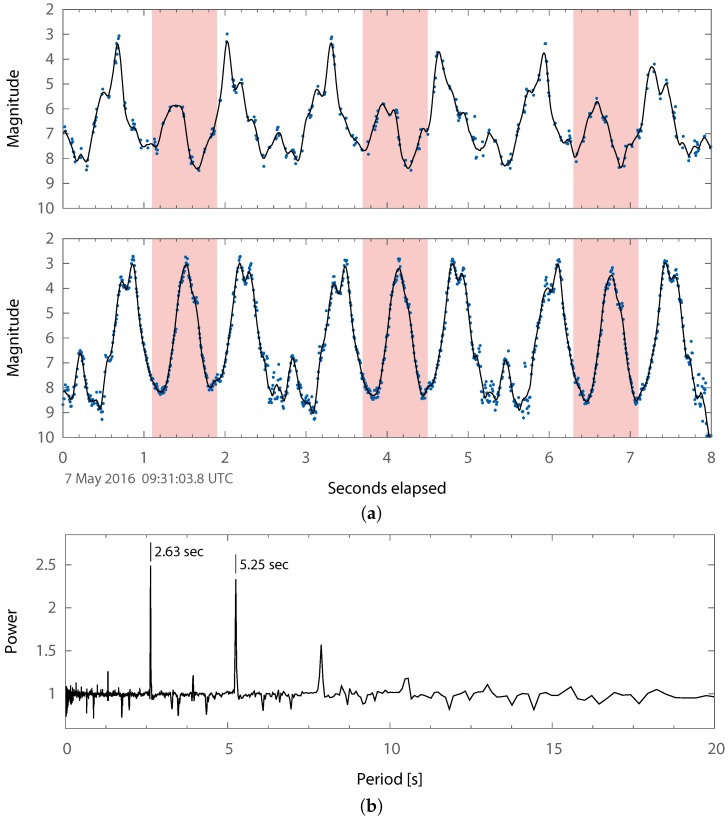
(**a**) the ASTRO-H parent body (Spacetrack ID 41337) observed after fragmentation event using identical OSCOM systems from Daytona Beach, FL, USA (top) and West Palm Beach, FL , USA (bottom). These observations used an integration time of 5 ms for an imaging cadence of 135 Hz. Some frames were dropped at the Daytona Beach site, causing occasional data gaps. The smoothing lines are 10 point centered moving averages. The line for the Daytona Beach site is drawn for a 10 ms resampled version of the data that was interpolated with the piecewise cubic Hermite interpolating polynomial (PCHIP) algorithm, but only the original data are shown as points. (**b**) Plavchan periodogram produced from a 1 min segment of the 7 May 2016 observation in West Palm Beach, FL, USA.

At an orbital altitude of about 575 km, ASTRO-H Object A was a bright source that allowed OSCOM to regularly record data at 100 Hz. Individual observations were obtained at rates as high as 350 Hz. From Figure 10, the object appears to have a primary flash period of about 2.6 s, but it is possible that the true object rotation rate is ∼5.2s given the high degree of symmetry. The periodogram in Figure 10b has nearly equal amounts of power at both of those periods.

The shape of the light curve observed from both sites is similar except for the difference in magnitude of the peaks highlighted in Figure 10a, and the ∼0.2s delay between corresponding features. Although we cannot be certain what specifically is causing this difference, in general, it is due to the difference in lighting geometry and aspect angle between the two sites. Further work is required to be able to estimate the object attitude using these differences.

## 7. Summary

With the increasing population of resident space objects in low Earth orbit, photometry is a crucial mission support and anomaly resolution tool for unresolved objects. It will especially be needed for object identification within a swarm of small satellites. The advent of CubeSats has made space accessible to all sorts of educational institutions and developing countries. Just as CubeSat mass appeal has been made possible by low cost of entry, so should photometric observations for mission support of small satellites. This paper has introduced the OSCOM electro-optics system for low cost optical observation of CubeSats and other small satellites. Its affordability and portable nature is crucial to allow users to acquire multiple systems and perform simultaneous observations from multiple sites, as we have demonstrated. The system is capable of performing various mission support duties as well as other space situational awareness tasks, such as observation of objects in GEO and simultaneous observation with different photometric filters for estimation of RSO material makeup. In the future, we hope to demonstrate a fully automated version of OSCOM for nightly multi-site observations.

Although the OSCOM system design should work for the majority of observatory sites, it is important to note that the specific components outlined above are not ideal for every site. For best photometric performance, especially for dim targets, it is necessary to match pixel scale with atmospheric seeing while maximizing the aperture. At sites with dark sky background, it is particularly important to have a low read noise detector to push the system to dimmer RSOs. Lastly, although flash periods can be extracted from light curves visually or by spectral analysis techniques, further work is required to estimate the attitude of an RSO, especially using multi-site simultaneous observations.

## Figures and Tables

**Table 1:** Components of the $10,000 OSCOM small satellite tracking system.

Purpose	Manufacturer	Model	Cost ($)
Telescope	Celestron	11 inch Rowe Ackermann Schmidt Astrograph	3500
Mount	Losmandy	G-11	3500
Imager	Allied Vision	Manta G-235 (Sony IMX174 CMOS)	1300
Computer	—	—	1000
Filters	Astrodon Photometrics	BVR	600
Peripherals	—	—	100

**Table 2:** OSCOM system specifications with the 11 inch Rowe Ackermann Schmidt Astrograph telescope and Manta G-235 imager.

Parameter	Value
Aperture diameter	279 mm
Focal length	620 mm
Pixel size	5.86 μm
Imager field of view	$1.04^{\circ }\times 0.65^{\circ }$
Pixel scale	1.95 arcsec/pix

## Abbreviations

The following abbreviations are used in this manuscript:

CCD	Charge-coupled device
CMOS	Complementary metal-oxide-semiconductor
COTS	Commercial off-the-shelf
FWHM	Full width half maximum
GEO	Geosynchronous Earth orbit
LEO	Low Earth orbit
NTP	Network time protocol
OSCOM	Optical tracking and spectral characterization of CubeSats for operational missions
QE	Quantum efficiency
RSO	Resident space object
SNR	Signal-to-noise ratio
SSA	Space situational awareness
TLE	Two-line element set
